# Complete mitochondrial genome of *Thalassiosira profunda* (Mediophyceae, Bacillariophyta)

**DOI:** 10.1080/23802359.2021.1916409

**Published:** 2021-04-26

**Authors:** Kuiyan Liu, Shuya Liu, Yang Chen, Feng Liu, Yongfang Zhao, Nansheng Chen

**Affiliations:** a CAS Key Laboratory of Marine Ecology and Environmental Sciences, Institute of Oceanology, Chinese Academy of Sciences, Qingdao, China; b Laboratory of Marine Ecology and Environmental Science, Qingdao National Laboratory for Marine Science and Technology, Qingdao, China; c School of Earth and Planetary, University of Chinese Academy of Sciences, Beijing, China; d Center for Ocean Mega-Science, Chinese Academy of Sciences, Qingdao, China; e Jiaozhou Bay National Marine Ecosystem Research Station, Institute of Oceanology, Chinese Academy of Sciences, Qingdao, China; f Department of Molecular Biology and Biochemistry, Simon Fraser University, Burnaby, Canada

**Keywords:** Diatoms, mitochondrial genome, *Thalassiosira profunda*

## Abstract

*Thalassiosira* is a species-rich genus with about 170 described species, many of which are harmful algal species with significant negative ecological impact. However, genome data of these species remain limited. In this study, the complete mitochondrial genome of *Thalassiosira profunda* (Hendey) Hasle 1973 was determined for the first time. The circular genome was 40,470 bp in length with GC content of 30.98%. It encodes 63 genes including 36 protein-coding genes (PCGs), 25 tRNA genes, and two rRNA genes. Phylogenetic analysis using concatenated PCGs suggested that *T. profunda* had a closer evolutionary relationship with *Skeletonema marinoi* of a different family (Skeletonemataceae) than *Thalassiosira pseudonana*, suggesting complex evolutionary relationship among species in these two families. Colinearity analysis also revealed fewer genome rearrangements between *T. profunda* and *S. marinoi* than that between *T. profunda* and *T. pseudonana*. This study suggests that mitochondrial genomes of many more species in the Thalassiosiraceae and Skeletonemataceae families are needed to disentangle the complex evolutionary relationships in the order of Thalassiosirales.

*Thalassiosira* (Mediophyceae, Bacillariophyta) is a species-rich genus with about 170 species described globally (Guiry and Guiry 2020) and about 50 species described in China (Li 2006). At least 10 *Thalassiosira* species, such as *Thalassiosira rotula*, *Thalassiosira diporocyclus*, and *Thalassiosira weissflogii*, have been found to form blooms with negative impact on environment (Li 2006; Li et al. 2013). Despite their important role in environment and ecology, molecular analysis of species in this genus has been limited. Here, we constructed the complete mitochondrial genome of *Thalassiosira profunda* (Hendey) Hasle 1973. The strain CNS00050 was isolated in water samples collected during an expedition to the Jiaozhou Bay (36°01.481′N, 120°17.202′E) in March 2019 onboard the research vehicle ‘Innovation’. The strain CNS00050 was confirmed to be *T. profunda* based on its morphological features and molecular sequences. The cells of CNS00050 were small, with diameters being 3–5 µm. Phylogenetic analysis of full-length 18S rDNA sequences indicated that the full-length 18S rDNA sequence of CNS00050 (MW205689) clustered with four *T. profunda* 18S rDNA sequences (KC284713, MN528652, MN528651, and MN528654) reported previously (Alverson 2016; Arsenieff et al. 2020). Another *T. profunda* full-length 18S rDNA sequence (AM235383) was clustered with *Thalassiosira nordenskioeldii*. However, this sequence was not supported by any published evidence. Similar phylogenetic analysis of other molecular markers including 28S rDNA D1-D2 regions (MW205747), *rbcL* (MW478286), and ITS (MW474850) all supported that the strain CNS00050 was *T. profunda*. Its specimen was deposited in the collection of marine algae in KLMEES of IOCAS (Nansheng Chen, chenn@qdio.ac.cn) under the voucher number CNS00050.

Illumina sequencing results of *T. profunda* were assembled into scaffolds using SPAdes v3.13.2 (Bankevich et al. 2012) and Platanus-allee v2.2.2 (Kajitani et al. 2019). Scaffolds of target mitochondrial genomes were selected from the assembly results using BLASTN v2.10.0. The mitochondrial genome sequence was examined using DOTTER v4.44.1 (Sonnhammer and Durbin 1995) and validated using the MEM algorithm of BWA v0.7.17 (Li and Durbin 2010). The alignments were visualized using IGV v2.8.12 (Robinson et al. 2011). Open reading frames (*orf*s) in the mitochondrial genome were first identified using Open Reading Frame Finder (ORF finder) (https://www.ncbi.nlm.nih.gov/orffinder) with ‘Genetic code: 4, ORF start codon: ‘ATG’ only’ selected. Protein-coding genes (PCGs) annotation was performed by using SmartBLAST (https://blast.ncbi.nlm.nih.gov/smartblast/) and BLASTP. tRNA genes were annotated using tRNAscan-SE 2.0 (Chan and Lowe 2019) with default setting. The locations of rRNAs were predicted by MFannot (https://megasun.bch.umontreal.ca/RNAweasel/) and determined by direct alignment with the mitogenomes of related species using MEGA X (Kumar et al. 2018) and BLASTN. The annotations were converted into genome maps by using OrganellarGenomeDRAW (OGDRAW) (Greiner et al. 2019).

The complete mitochondrial genome of *T. profunda* (GenBank accession number: MW013551) is 40,470 bp in size with GC content of 30.98%. It encodes 63 genes including 36 PCGs, 25 tRNA genes, and two rRNA genes. Among the 36 PCGs, 34 genes start with the canonical ATG start codons, *nad11* with TTG, and *atp8* with ATT. Most genes have canonical stop codons TAA (31 of 36 genes), with five genes having TAG as stop codons. The 25 tRNA genes, ranging in length from 72 bp to 89 bp, have typical cloverleaf secondary structures. No introns were found in the *T. profunda* mitochondrial genome.

Maximum-likelihood (ML) phylogenetic tree (Figure 1) was constructed using tandem amino acid sequences of 31 common genes including *atp6*, *8*, *9*; *cob*; *cox1*, *2*, *3*; *nad1*–*7*, *4L*, *9*, *11*; *rpl2*, *5*, *6*, *14*, *16*; *rps3*, *4*, *8*, *10*, *11*, *13*, *14*, *19*; and *tat*C, from 35 publicly diatom mitochondrial genomes using IQtree v1.6.12 (Trifinopoulos et al. 2016) with 1000 bootstrap alignments. Mitochondrial genomes of two Oomycota species *Phytophthora ramorum* (EU427470) and *Saprolegnia ferax* (NC_005984) were used as out-group taxa. The results demonstrated that species fell nicely into three clades corresponding to three classes of the phylum Bacillariophyta including Coscinodiscophyceae, Mediophyceae, and Bacillariophyceae. *T. profunda* was grouped with *Skeletonema marinoi* and *T. pseudonana* with strong support. *T. profunda* of the family Thalassiosiraceae showed closer evolutionary relationship with *S. marinoi* of the family Skeletonemataceae than that with *T. pseudonana*, which were different families in the order Thalassiosirales (Stoermer 2003). Furthermore, colinearity analysis of the mitochondrial genomes of three species *T. profunda*, *T. pseudonana*, and *S. marinoi* identified a single inversion event involving a single gene *atp6* between *T. profunda* and *S. marinoi*, while identified an inversion event involving *atp6* plus a translocation event involving two genes *cox2*-*cox3* between *T. profunda* and *T. pseudonana*, also suggesting higher similarity between *T. profunda* and *S. marinoi*. These results were consistent with findings from a recent study suggesting that *T. pseudonana* should be classified as a species of another genus *Cyclotella* (Alverson et al. 2011). Thus, the mitochondrial genome of *T. profunda* likely represents that first mitochondrial genome of *Thalassiosira*. The complete mitochondrial genomes of more species in *Thalassiosira* and related genus will help to clarify the evolutionary relationships and classification of the order Thalassiosirales.

**Figure 1.**
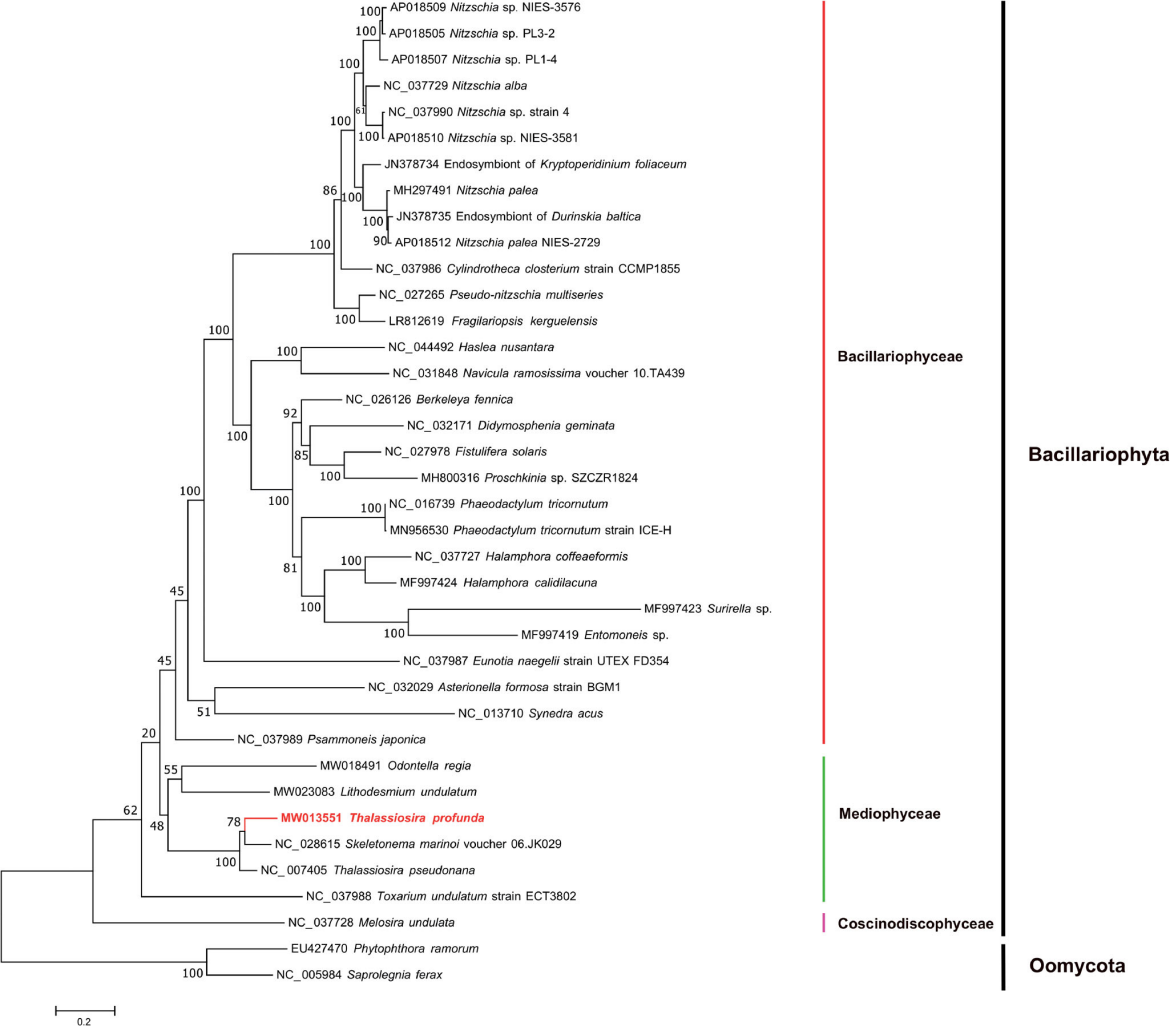
Maximum-likelihood (ML) phylogenetic tree based on tandem amino acid sequences of 31 common genes from 35 publicly diatom mitochondrial genomes, and *Phytophthora ramorum* (EU427470) and *Saprolegnia ferax* (NC_005984) in Oomycota were used as out-group taxa. The numbers beside branch nodes are the percentage of 1000 bootstrap values.

## Data Availability

The genome sequence data that support the findings of this study are openly available in GenBank of NCBI at https://www.ncbi.nlm.nih.gov/nuccore/MW013551, under the accession no. MW013551. The associated BioProject, SRA, and Bio-Sample numbers are PRJNA684688 (https://www.ncbi.nlm.nih.gov/bioproject/PRJNA684688), SRR13245496 (https://www.ncbi.nlm.nih.gov/sra/SRR13245496), and SAMN17065834 (https://www.ncbi.nlm.nih.gov/biosample/SAMN17065834/), respectively.
